# *United European Gastroenterology* Journal: Striving for High‐Quality Clinical Guidelines

**DOI:** 10.1002/ueg2.70225

**Published:** 2026-05-05

**Authors:** Mohamed G. Shiha, Yasuko Maeda

**Affiliations:** ^1^ Division of Clinical Medicine School of Medicine and Population Health University of Sheffield Sheffield UK; ^2^ Department of Gastroenterology University Hospitals of Leicester Leicester UK; ^3^ Department of Surgery Queen Elizabeth University Hospital Glasgow UK; ^4^ School of Medicine, Dentistry and Nursing University of Glasgow Glasgow UK

## 1 Introduction

Over the past decade, *United European Gastroenterology* (UEG) Journal has proudly served as the home for European clinical guidelines. Clinicians across Europe and around the world rely on these comprehensive documents to make everyday clinical decisions. Recognising this responsibility, UEG started several initiatives to improve the quality of clinical guidelines. These efforts include endorsing the GRADE methodology, developing a robust framework, and launching accessible UEG ‘Gutflix’ courses to educate teams on guideline development [1]. Yet, a recent evaluation of 16 clinical guidelines published in *UEG Journal* between 2020 and 2022 showed a high variability in quality, with critical shortcomings in certain domains, such as clinical applicability, where most guidelines lacked evaluation of potential barriers or practical implementation tools. Moreover, stakeholders' roles were frequently ambiguous and individual recommendations often lacked a clear purpose [2]. We asked our readership, ‘What constitutes high‐quality clinical guidelines?’, and the answer was definite: Clinicians want clear, evidence‐based, actionable recommendations in concise and accessible formats [3].

## 2 Moving Away From ‘Expert Consensus’

To deliver this mandate, we must move away from the historical reliance on ‘expert consensus’. While an expert's opinion is valuable, it is inherently subjective, prone to bias and may not reflect the best available evidence or the lack of it. GRADE is a globally established methodology adopted by more than 100 organisations, including the World Health Organisation (WHO). For *UEG* *Journal*, nominal use of the GRADE framework is no longer sufficient. Strict adherence to its core principles and the execution of its steps without ad hoc modifications are imperative prerequisites for publication. Core GRADE is based on two fundamental principles: judging the certainty of evidence for each important outcome and moving from evidence to recommendations that have both a direction (for or against) and a strength (strong or conditional/weak) [4]. The transition from evidence to recommendation is achieved only after systematically weighing clinical benefits against potential harms and considering resource implications, feasibility, acceptability, and patient values.

## 3 The Role of the Methodologist

Relying exclusively on busy full‐time clinicians to navigate this complex process is an outdated strategy. Expert clinicians rarely have the formal training or the dedicated time to perform rigorous systematic literature searches, critically appraise evidence and synthesise findings in a transparent and methodologically robust manner. To achieve this level of methodological excellence, the inclusion of a dedicated, qualified methodologist is no longer optional. The methodologist should be involved from the early stages of protocol development to ensure that the guideline questions are appropriately structured based on the relevant population, interventions, comparisons and outcomes of potential importance to patients (PICO) [4]. They should also lead the planning and conduct of systematic literature reviews, oversee standardised data extraction, and support the formal assessment of evidence certainty. Overall, their involvement is essential to ensure consistent adherence to the GRADE Evidence‐to‐Decision framework that can lead to clear, transparent, and justifiable recommendations [5].

## 4 Precision and Applicability

A consistent piece of feedback from clinicians who use our guidelines is that they are often too long, too complex, and too difficult to apply in clinical practice [3]. Guidelines that attempt to cover every conceivable clinical question about a condition or a disease within a single document overwhelm the reader with information that may not be relevant and take years to produce, risking being outdated by the time they are published. Guideline panels should limit their scope to a manageable number of PICO questions that are genuinely critical (e.g., 10–15 PICOs). This focused approach allows for a timely, up‐to‐date literature search and a realistic guideline development plan. This also ensures that all resulting recommendations are highly relevant and actionable. Importantly, guidelines should not include irrelevant background information and detailed literature reviews that are not linked to actionable recommendations.

Guidelines have traditionally been developed by academic sub‐specialists, who may fail to account for the practical realities and limitations outside tertiary centres. To ensure recommendations are applicable across the entire healthcare system, panels should include frontline clinicians, including those from primary care, alongside broader system stakeholders (e.g., regulatory agencies, hospital managers) who can provide insight into the cost, resource allocation and logistical feasibility required by the GRADE Evidence‐to‐Decision framework. The process of selecting and including panel members must be entirely open and transparent.

## 5 The Missing Stakeholder

The ultimate goal of clinical guidelines is to improve the quality of patient care and patient outcomes. However, patients are rarely given a seat at the table where these outcomes are defined and recommendations are made. Adherence to the GRADE framework requires an assessment of patient values and preferences. Yet, this aspect is often entirely overlooked or inferred indirectly from the clinicians' opinions. Excluding the patient voice risks producing guidelines that are disconnected from the lived realities of those they are meant to treat. To provide high‐quality clinical guidelines, patients and their representatives must be involved from the early stages of guideline development, including the formulation of key clinical questions and rating the importance of outcomes [6]. Their active participation is also critical during the interpretation of evidence and the formulation of recommendations to ensure they are aligned with patient values and real‐world priorities.

## 6 The Future of UEG Guidelines

*UEG* *Journal* is striving to publish high‐quality clinical guidelines that are relevant, precise and evidence‐based. Achieving these high standards requires strict adherence to the GRADE framework and a fundamental shift in our publication model. The traditional approach of producing lengthy, exhaustive documents is no longer sustainable in a rapidly evolving medical landscape. This model places substantial demands on the authors, wastes valuable resources, and often results in guidelines that can quickly become outdated [7]. The future is ‘living guidelines’ and focused updates [8]. Guideline development groups can use this dynamic methodology to regularly monitor the literature and swiftly integrate practice‐changing evidence as soon as it emerges. This continuous cycle of literature appraisal ensures that our guidelines remain a timely, actionable and reliable resource for clinicians. Our journal welcomes initiatives to provide focused updates addressing single or limited clinical questions based on the publication of landmark studies or a significant shift in the evidence base.

## Conflicts of Interest

The authors have nothing to report.

## Data Availability

Data sharing not applicable to this article as no datasets were generated or analysed during the current study.
